# *N*-(3-Chloro­phen­yl)-*N*′-(3-methyl­phen­yl)succinamide

**DOI:** 10.1107/S1600536811028121

**Published:** 2011-07-23

**Authors:** B. S. Saraswathi, Sabine Foro, B. Thimme Gowda

**Affiliations:** a Department of Chemistry, Mangalore University, Mangalagangotri 574 199, Mangalore, India; b Institute of Materials Science, Darmstadt University of Technology, Petersenstrasse 23, D-64287 Darmstadt, Germany

## Abstract

The asymmetric unit of the title compound, C_17_H_17_ClN_2_O_2_, contains one half-mol­ecule with a center of inversion at the mid-point of the central C—C bond. The amide N—H group is *anti* to the *meta*-chloro/methyl groups in the adjacent benzene rings. The dihedral angle between the benzene ring and the NH—C(O)—CH_2_ segment is 43.5 (1)°. In the crystal, inter­molecular N—H⋯O hydrogen bonds link the mol­ecules into chains along the *a* axis. The methyl group and the Cl atom occupy the same position and were treated in a disorder model with site-occupation factors of 0.5 each.

## Related literature

For our studies on the effects of substituents on the structures of *N*-(ar­yl)-amides, see: Bhat & Gowda (2000 ▶); Gowda *et al.* (2007 ▶); Saraswathi *et al.* (2011**a* ▶,b* ▶) and on the structures of *N*-(ar­yl)-methane­sulfonamides, see: Jayalakshmi & Gowda (2004 ▶). For similar structures, see: Pierrot *et al.* (1984 ▶). For restrained geometry, see: Nardelli (1999 ▶).
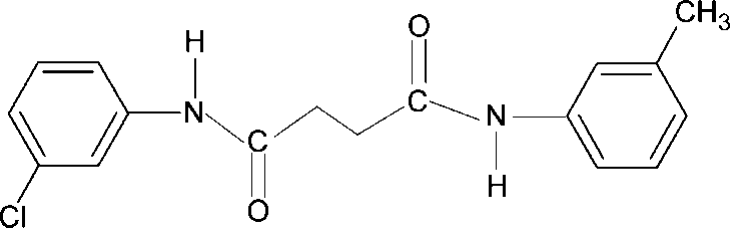


## Experimental

### 

#### Crystal data

C_17_H_17_ClN_2_O_2_
*M* *_r_* = 316.78
Triclinic, 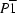

*a* = 4.840 (1) Å
*b* = 5.560 (1) Å
*c* = 14.752 (3) Å
α = 93.47 (2)°
β = 91.39 (2)°
γ = 97.71 (2)°
*V* = 392.46 (13) Å^3^
*Z* = 1
Mo *K*α radiation
μ = 0.25 mm^−1^
*T* = 293 K
0.44 × 0.20 × 0.08 mm

#### Data collection

Oxford Diffraction Xcalibur diffractometer with Sapphire CCD detector
Absorption correction: multi-scan (*CrysAlis RED*; Oxford Diffraction, 2009 ▶) *T*_min_ = 0.897, *T*_max_ = 0.980
2451 measured reflections
1567 independent reflections
1249 reflections with *I* > 2σ(*I*)
*R*_int_ = 0.010

#### Refinement

*R*[*F* ^2^ > 2σ(*F* ^2^)] = 0.050
*wR*(*F* ^2^) = 0.130
*S* = 0.99
1567 reflections
110 parameters
14 restraints
H-atom parameters constrained
Δρ_max_ = 0.17 e Å^−3^
Δρ_min_ = −0.17 e Å^−3^

### 

Data collection: *CrysAlis CCD* (Oxford Diffraction, 2009 ▶); cell refinement: *CrysAlis RED* (Oxford Diffraction, 2009 ▶); data reduction: *CrysAlis RED*; program(s) used to solve structure: *SHELXS97* (Sheldrick, 2008 ▶); program(s) used to refine structure: *SHELXL97* (Sheldrick, 2008 ▶); molecular graphics: *PLATON* (Spek, 2009 ▶); software used to prepare material for publication: *SHELXL97*.

## Supplementary Material

Crystal structure: contains datablock(s) I, global. DOI: 10.1107/S1600536811028121/wm2508sup1.cif

Structure factors: contains datablock(s) I. DOI: 10.1107/S1600536811028121/wm2508Isup2.hkl

Supplementary material file. DOI: 10.1107/S1600536811028121/wm2508Isup3.cml

Additional supplementary materials:  crystallographic information; 3D view; checkCIF report

## Figures and Tables

**Table 1:** Hydrogen-bond geometry (Å, °)

*D*—H⋯*A*	*D*—H	H⋯*A*	*D*⋯*A*	*D*—H⋯*A*
N1—H1⋯O1^i^	0.86	2.05	2.894 (2)	168

Symmetry code: (i) 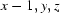.

**Fig. 1.**
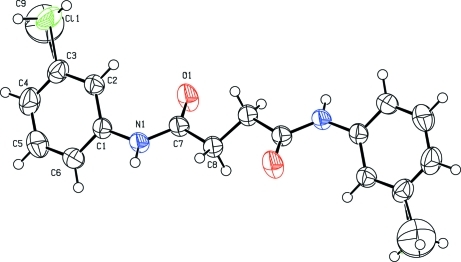
Molecular structure of the title compound, showing the atom labelling scheme and displacement ellipsoids are drawn at the 50% probability level.

**Fig. 2.**
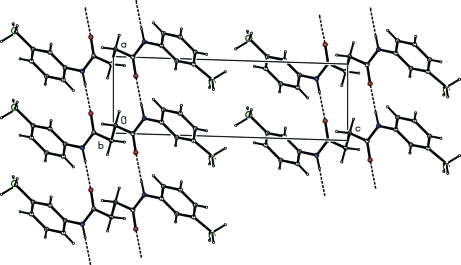
Molecular packing of the title compound with hydrogen bonding shown as dashed lines.

## supplementary crystallographic information

### Comment

The amide and sulfonamide moieties are important constituents of many biologically significant compounds. As part of our studies on the substituent effects on the structures of this class of compounds (Bhat & Gowda, 2000; Gowda *et al.*, 2007; Jayalakshmi & Gowda, 2004; Saraswathi *et al.*, 2011**a*,b*), in the present work, the structure of *N*-(3-chlorophenyl),*N*-(3-methylphenyl)- succinamide, (I), has been determined (Fig.1). The asymmetric unit of (I) contains half a molecule with a center of inversion at the mid-point of the central C—C bond, similar to that observed in bis(2-chlorophenylaminocarbonylmethyl)disulfide, (II), (Pierrot *et al.*, 1984), *N*,*N*-bis(3-chlorophenyl)-succinamide, (III), (Saraswathi *et al.*, 2011*a*) and *N*,*N*-bis(3-methylphenyl)-succinamide dihydrate, (IV), (Saraswathi *et al.*, 2011*b*).

The conformations of the amide O atoms are *anti* to the H atoms attached to the adjacent C atoms. Further, the conformations of the N—H bonds in the amide fragments are *anti* to the *meta*-chloro/methyl groups in the adjacent benzene rings, similar to the *anti* conformations observed with respect to the *meta*-chloro groups in (III) and *meta*-methyl groups in (IV).

Further, the C1—N1—C7—C8 and C1a—N1a—C7a—C8a segments in (I) are nearly planar and so also the C1—N1—C7—O1 and C1a—N1a—C7a—O1a segments, similar to those observed in (III) and (IV). The torsion angles of C2—C1—N1—C7 and C6—C1—N1—C7 are -43.2 (4)° and 138.6 (3)°, in contrast to the values of -35.0 (3)° and 147.5 (2)° in (III), and 5.4 (9)° and -173.6 (6)° in (IV).

The dihedral angle between the benzene ring and the NH—C(O)—CH_2_ segment is 43.5 (1)°, compared to the values of 62.1 (2)° in (III) and 5.6 (4)° in (IV).

The packing of the molecules in the crystal is accomplished by N—H···O hydrogen bonds (Table 1) that lead is shown in Fig. 2.

### Experimental

Succinic anhydride (0.01 mol) in toluene (25 ml) was treated dropwise with 3-chloroaniline (0.01 mol) also in toluene (20 ml) with constant stirring. The resulting mixture was stirred for one hour and set aside for an additional hour at room temperature for completion of the reaction. The mixture was then treated with dilute hydrochloric acid to remove unreacted 3-chloroaniline. The resultant solid *N*-(3-chlorophenyl)-succinamic acid was filtered under suction and washed thoroughly with water to remove the unreacted succinic anhydride and succinic acid. The compound was recrystallized to a constant melting point from ethanol. The purity of the compound was checked by elemental analysis and characterized by its infrared and NMR spectra.

The *N*-(3-chlorophenyl)succinamic acid obtained was then treated with phosphorous oxychloride and excess of 3-methylaniline at room temperature with constant stirring. The resultant mixture was stirred for 4 h, kept aside for additional 6 h for completion of the reaction and poured slowly into crushed ice with constant stirring. It was kept aside for a day. The resultant solid, *N*-(3-chlorophenyl), *N*-(3-methylphenyl)-succinamide was filtered under suction, washed thoroughly with water, dilute sodium hydroxide solution and finally with water. It was recrystallized to a constant melting point from a mixture of acetone and toluene (3:1 *v*/*v*). The compound was characterized by its infrared and NMR spectra.

Prism-like colorless single crystals used in X-ray diffraction studies were grown in a mixture of acetone and toluene (3:1 *v*/*v*) at room temperature.

### Refinement

The H atoms were positioned with idealized geometry using a riding model with C—H = 0.93 Å for aromatic, C—H = 0.97 Å for methylene and N—H = 0.86 Å for amide H atoms and were refined with isotropic displacement parameters, set to 1.2×*U*_eq_ of the parent atom. Atoms C9 and Cl1 occupy the same position. The disorder was treated by using a split-atom model. The corresponding site-occupation factors were fixed to 0.50:0.50. The U^ij^ components of these atoms were restrained to approximate isotropic behavior (Nardelli, 1999), the bond lenghts C3—C9 and C3—Cl1 were restrained.

### Crystal data

**Table:**

C_17_H_17_ClN_2_O_2_	*Z* = 1
*M**_r_* = 316.78	*F*(000) = 166
Triclinic, *P*1	*D*_x_ = 1.340 Mg m^−^^3^
Hall symbol: -P 1	Mo *K*α radiation, λ = 0.71073 Å
*a* = 4.840 (1) Å	Cell parameters from 1094 reflections
*b* = 5.560 (1) Å	θ = 2.8–27.7°
*c* = 14.752 (3) Å	µ = 0.25 mm^−^^1^
α = 93.47 (2)°	*T* = 293 K
β = 91.39 (2)°	Prism, colourless
γ = 97.71 (2)°	0.44 × 0.20 × 0.08 mm
*V* = 392.46 (13) Å^3^	

### Data collection

**Table:**

Oxford Diffraction Xcalibur diffractometer with Sapphire CCD detector	1567 independent reflections
Radiation source: fine-focus sealed tube	1249 reflections with *I* > 2σ(*I*)
graphite	*R*_int_ = 0.010
Rotation method data acquisition using ω scans	θ_max_ = 26.3°, θ_min_ = 2.8°
Absorption correction: multi-scan (*CrysAlis RED*; Oxford Diffraction, 2009)	*h* = −6→5
*T*_min_ = 0.897, *T*_max_ = 0.980	*k* = −6→6
2451 measured reflections	*l* = −18→17

### Refinement

**Table:**

Refinement on *F*^2^	Primary atom site location: structure-invariant direct methods
Least-squares matrix: full	Secondary atom site location: difference Fourier map
*R*[*F*^2^ > 2σ(*F*^2^)] = 0.050	Hydrogen site location: inferred from neighbouring sites
*wR*(*F*^2^) = 0.130	H-atom parameters constrained
*S* = 0.99	*w* = 1/[σ^2^(*F*_o_^2^) + (0.0501*P*)^2^ + 0.2642*P*] where *P* = (*F*_o_^2^ + 2*F*_c_^2^)/3
1567 reflections	(Δ/σ)_max_ < 0.001
110 parameters	Δρ_max_ = 0.17 e Å^−^^3^
14 restraints	Δρ_min_ = −0.17 e Å^−^^3^

### Special details

**Table:**

Geometry. All e.s.d.'s (except the e.s.d. in the dihedral angle between two l.s. planes) are estimated using the full covariance matrix. The cell e.s.d.'s are taken into account individually in the estimation of e.s.d.'s in distances, angles and torsion angles; correlations between e.s.d.'s in cell parameters are only used when they are defined by crystal symmetry. An approximate (isotropic) treatment of cell e.s.d.'s is used for estimating e.s.d.'s involving l.s. planes.
Refinement. Refinement of *F*^2^ against ALL reflections. The weighted *R*-factor *wR* and goodness of fit *S* are based on *F*^2^, conventional *R*-factors *R* are based on *F*, with *F* set to zero for negative *F*^2^. The threshold expression of *F*^2^ > σ(*F*^2^) is used only for calculating *R*-factors(gt) *etc*. and is not relevant to the choice of reflections for refinement. *R*-factors based on *F*^2^ are statistically about twice as large as those based on *F*, and *R*- factors based on ALL data will be even larger.

### Fractional atomic coordinates and isotropic or equivalent isotropic displacement parameters (Å^2^)

**Table:**

	*x*	*y*	*z*	*U*_iso_*/*U*_eq_	Occ. (<1)
O1	0.2418 (3)	0.7102 (3)	−0.09593 (13)	0.0651 (6)	
N1	−0.2109 (3)	0.5789 (3)	−0.13211 (12)	0.0417 (5)	
H1	−0.3807	0.5949	−0.1204	0.050*	
C1	−0.1722 (4)	0.4107 (4)	−0.20578 (14)	0.0384 (5)	
C2	0.0217 (4)	0.4712 (4)	−0.27063 (14)	0.0445 (5)	
H2	0.1310	0.6226	−0.2657	0.053*	
C3	0.0541 (5)	0.3084 (5)	−0.34254 (15)	0.0520 (6)	
C4	−0.1059 (6)	0.0831 (5)	−0.34971 (18)	0.0630 (7)	
H4A	−0.0831	−0.0283	−0.3977	0.076*	
C5	−0.2988 (6)	0.0250 (5)	−0.2854 (2)	0.0659 (7)	
H5A	−0.4070	−0.1269	−0.2904	0.079*	
C6	−0.3364 (5)	0.1866 (4)	−0.21353 (16)	0.0502 (6)	
H6	−0.4703	0.1453	−0.1709	0.060*	
C7	−0.0058 (4)	0.7190 (4)	−0.08329 (14)	0.0414 (5)	
C8	−0.0999 (4)	0.8842 (4)	−0.00851 (15)	0.0441 (5)	
H8A	−0.1160	0.7990	0.0470	0.053*	
H8B	−0.2828	0.9240	−0.0249	0.053*	
C9	0.251 (3)	0.364 (4)	−0.4221 (10)	0.155 (8)	0.50
H9A	0.3567	0.5224	−0.4105	0.185*	0.50
H9B	0.1426	0.3602	−0.4776	0.185*	0.50
H9C	0.3763	0.2442	−0.4274	0.185*	0.50
Cl1	0.2883 (5)	0.3904 (5)	−0.42162 (12)	0.0742 (6)	0.50

### Atomic displacement parameters (Å^2^)

**Table:**

	*U*^11^	*U*^22^	*U*^33^	*U*^12^	*U*^13^	*U*^23^
O1	0.0252 (8)	0.0862 (13)	0.0794 (12)	0.0103 (7)	0.0031 (7)	−0.0380 (10)
N1	0.0249 (8)	0.0507 (11)	0.0484 (10)	0.0059 (7)	0.0052 (7)	−0.0101 (8)
C1	0.0295 (9)	0.0447 (12)	0.0415 (11)	0.0094 (8)	−0.0013 (8)	−0.0030 (9)
C2	0.0366 (11)	0.0497 (13)	0.0457 (12)	0.0028 (9)	0.0031 (9)	−0.0039 (9)
C3	0.0458 (12)	0.0695 (16)	0.0415 (12)	0.0152 (11)	0.0031 (10)	−0.0051 (11)
C4	0.0707 (17)	0.0637 (17)	0.0532 (14)	0.0147 (13)	0.0009 (12)	−0.0188 (12)
C5	0.0738 (18)	0.0468 (14)	0.0717 (17)	−0.0039 (12)	−0.0025 (14)	−0.0111 (12)
C6	0.0462 (12)	0.0493 (13)	0.0526 (13)	−0.0017 (10)	0.0059 (10)	−0.0003 (10)
C7	0.0283 (10)	0.0485 (12)	0.0471 (12)	0.0073 (8)	0.0046 (8)	−0.0065 (10)
C8	0.0302 (10)	0.0550 (13)	0.0458 (11)	0.0071 (9)	0.0048 (8)	−0.0114 (10)
C9	0.149 (9)	0.162 (9)	0.152 (9)	0.019 (5)	0.009 (5)	0.006 (5)
Cl1	0.0639 (9)	0.1109 (15)	0.0466 (7)	0.0093 (9)	0.0242 (7)	−0.0080 (8)

### Geometric parameters (Å, °)

**Table:**

O1—C7	1.224 (2)	C4—H4A	0.9300
N1—C7	1.342 (3)	C5—C6	1.380 (3)
N1—C1	1.423 (3)	C5—H5A	0.9300
N1—H1	0.8591	C6—H6	0.9300
C1—C6	1.382 (3)	C7—C8	1.510 (3)
C1—C2	1.381 (3)	C8—C8^i^	1.507 (4)
C2—C3	1.378 (3)	C8—H8A	0.9700
C2—H2	0.9300	C8—H8B	0.9700
C3—C4	1.379 (4)	C9—H9A	0.9600
C3—Cl1	1.686 (3)	C9—H9B	0.9600
C3—C9	1.550 (9)	C9—H9C	0.9600
C4—C5	1.369 (4)		
			
C7—N1—C1	125.39 (16)	C6—C5—H5A	119.2
C7—N1—H1	118.5	C5—C6—C1	119.0 (2)
C1—N1—H1	116.1	C5—C6—H6	120.5
C6—C1—C2	119.8 (2)	C1—C6—H6	120.5
C6—C1—N1	119.33 (19)	O1—C7—N1	122.90 (18)
C2—C1—N1	120.85 (19)	O1—C7—C8	121.54 (18)
C3—C2—C1	120.4 (2)	N1—C7—C8	115.51 (16)
C3—C2—H2	119.8	C7—C8—C8^i^	112.1 (2)
C1—C2—H2	119.8	C7—C8—H8A	109.2
C2—C3—C4	120.0 (2)	C8^i^—C8—H8A	109.2
C2—C3—Cl1	119.1 (2)	C7—C8—H8B	109.2
C4—C3—Cl1	120.9 (2)	C8^i^—C8—H8B	109.2
C2—C3—C9	124.4 (7)	H8A—C8—H8B	107.9
C4—C3—C9	115.4 (7)	C3—C9—H9A	109.5
Cl1—C3—C9	5.9 (7)	C3—C9—H9B	109.5
C5—C4—C3	119.2 (2)	H9A—C9—H9B	109.5
C5—C4—H4A	120.4	C3—C9—H9C	109.5
C3—C4—H4A	120.4	H9A—C9—H9C	109.5
C4—C5—C6	121.6 (2)	H9B—C9—H9C	109.5
C4—C5—H5A	119.2		
			
C7—N1—C1—C6	138.7 (2)	C9—C3—C4—C5	−175.8 (9)
C7—N1—C1—C2	−43.0 (3)	C3—C4—C5—C6	−0.1 (4)
C6—C1—C2—C3	−0.6 (3)	C4—C5—C6—C1	−1.0 (4)
N1—C1—C2—C3	−178.9 (2)	C2—C1—C6—C5	1.3 (3)
C1—C2—C3—C4	−0.5 (4)	N1—C1—C6—C5	179.6 (2)
C1—C2—C3—Cl1	178.8 (2)	C1—N1—C7—O1	−2.4 (4)
C1—C2—C3—C9	175.8 (9)	C1—N1—C7—C8	179.8 (2)
C2—C3—C4—C5	0.8 (4)	O1—C7—C8—C8^i^	32.9 (4)
Cl1—C3—C4—C5	−178.4 (2)	N1—C7—C8—C8^i^	−149.2 (2)

Symmetry codes: (i) −*x*, −*y*+2, −*z*.

### Hydrogen-bond geometry (Å, °)

**Table:**

*D*—H···*A*	*D*—H	H···*A*	*D*···*A*	*D*—H···*A*
N1—H1···O1^ii^	0.86	2.05	2.894 (2)	168.

Symmetry codes: (ii) *x*−1, *y*, *z*.
